# The Tumbling Bullet: Subacute Intestinal Obstruction due to a Retained Bullet

**DOI:** 10.7759/cureus.9844

**Published:** 2020-08-18

**Authors:** Anupam K Gupta, Blake Edwards

**Affiliations:** 1 Minimally Invasive Surgery, University of Miami Hospital, Miami, USA; 2 General Surgery, Boca Raton Regional Hospital, Florida Atlantic University, Boca Raton, USA

**Keywords:** gunshot injury to abdomen, damage control surgery, impacted bullet, gall stone ileus, intermittent obstruction

## Abstract

A 29-year-old female presented with multiple gunshot wounds to the back and bilateral lower extremities. The patient underwent an exploratory laparotomy with small-bowel resection of two segments with primary stapled anastomosis and partial nephrectomy. The postoperative course showed prolonged intermittent bowel obstruction secondary to the bullet, which lodged in the distal ileum. The patient eventually passed the bullet; it, however, led to a delay in recovery.

## Introduction

In trauma, penetrating injury in the form of a stab or gunshot is common, with the most common organ affected being small bowel (50%) [1]. In intraperitoneal injury, the patient undergoes an emergency exploratory laparotomy to control life-threatening injuries like bleeding, and restore bowel continuity [2-6]. Exploratory laparotomy for gunshot wounds of the abdomen does not prioritize the removal of the foreign body projectile unless it is directly in the field or causing life-threatening hemorrhage [6-10]. We report an unusual case of a bullet left inside, which caused prolonged intermittent obstruction postoperatively.

## Case presentation

**Figure 1:**
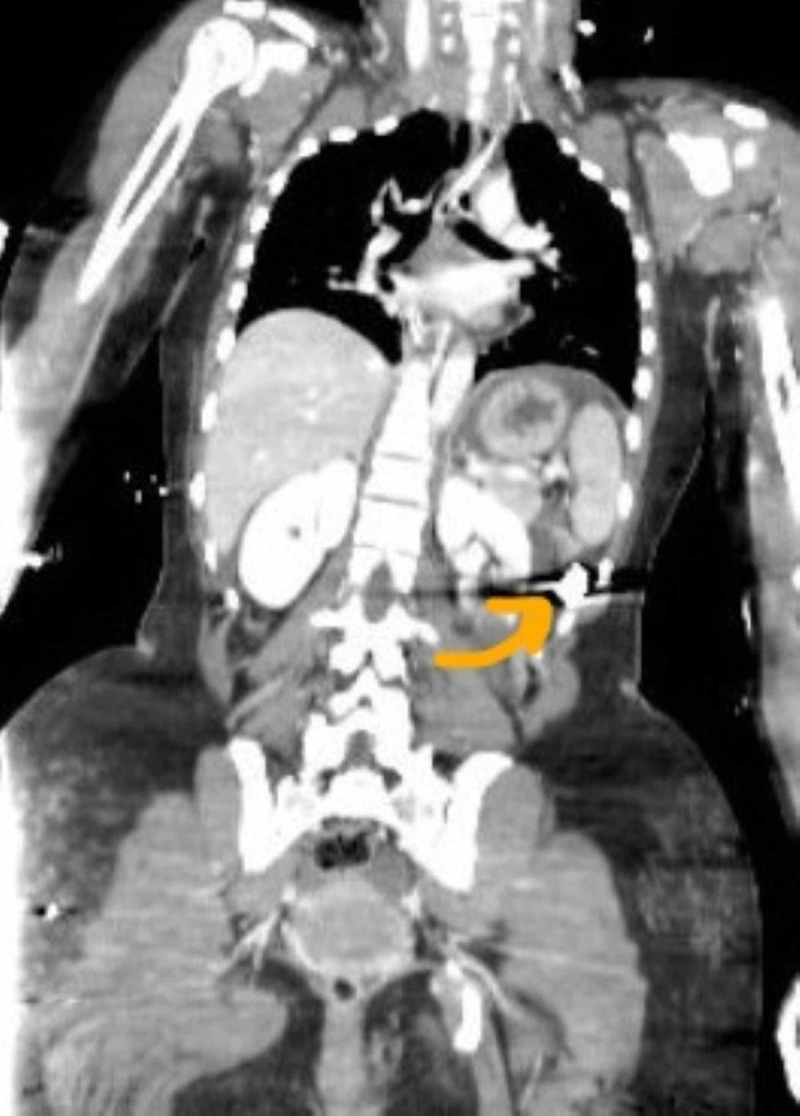
Bullet fragment in the left upper quadrant

A 29-year-old lady with no medical history presented after multiple gunshot wounds to the back, abdomen, and bilateral lower extremities. On arrival to the emergency room, the patient was on hardboard with a cervical collar, and the Glasgow coma score was 8 (eye opening in response to pain, the verbal response being incomprehensible, the motor response being flexion, and withdrawal from pain). Vital signs were significant for tachycardia to 115 beats/min, sinus rhythm, and blood pressure of 100/60 mm Hg, saturation 95% on room air. Under the primary survey, the patient was intubated and resuscitated with blood. In the secondary survey, there were gunshot wounds to the back and bilateral lower limbs. Bilateral lower limb,vascular examination showed palpable pulses with no hemorrhage. Once stabilized, the patient had a computed tomography study of the head, chest, abdomen, pelvis, and spine. Computed tomography was positive for the presence of the bullet in the left upper quadrant of the abdomen with pneumoperitoneum (Figure 1).

The patient underwent midline exploratory laparotomy. Significant injuries were several small bowel defects and left kidney lower pole injury. The patient underwent jejunal and ileal bowel resection with primary stapled anastomosis, and a partial nephrectomy of the left lower pole of the kidney was performed. The bullet fragment was not actively looked for and, at the time, assumed to be buried in retroperitoneum. The wounds on the limbs involved soft tissue and were treated with debridement and dressing.

**Figure 2:**
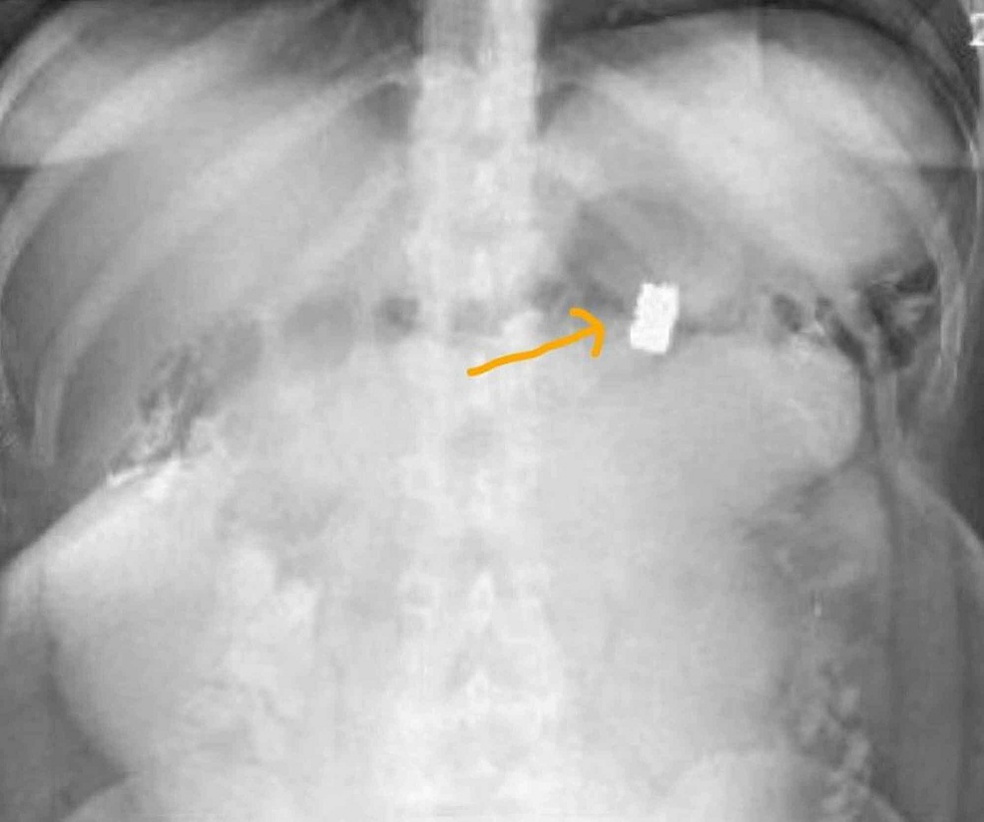
Left upper quadrant position of the bullet

**Figure 3:**
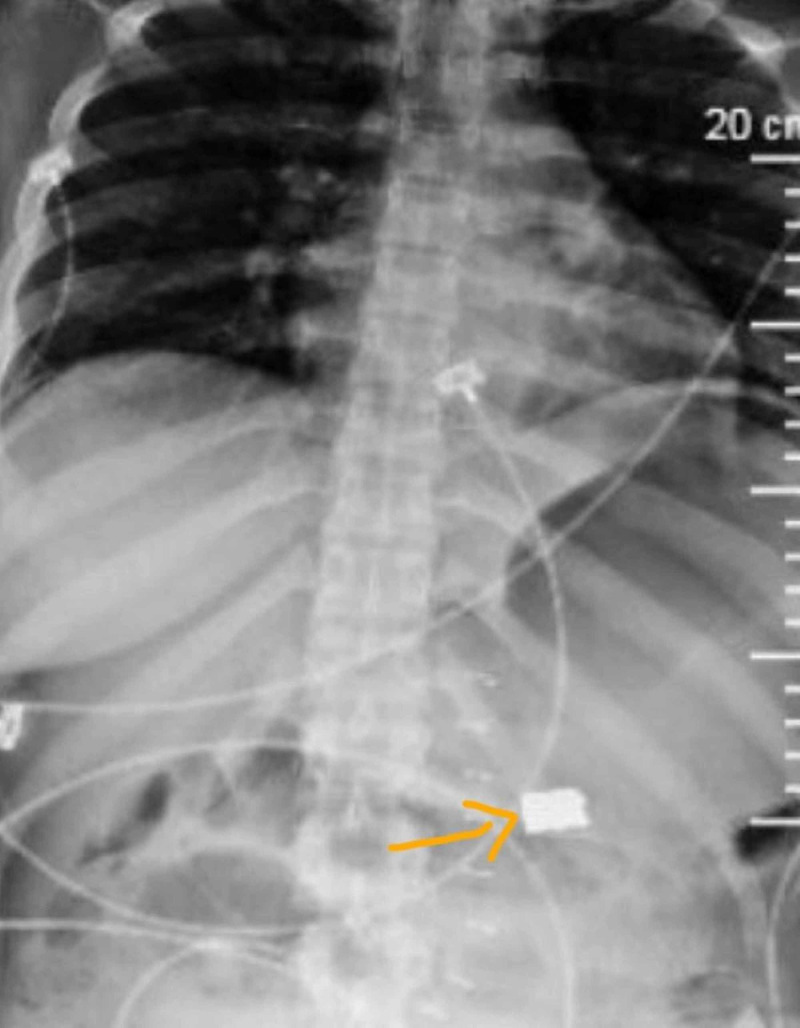
Progression in the position of the bullet, postoperative day 5

**Figure 4:**
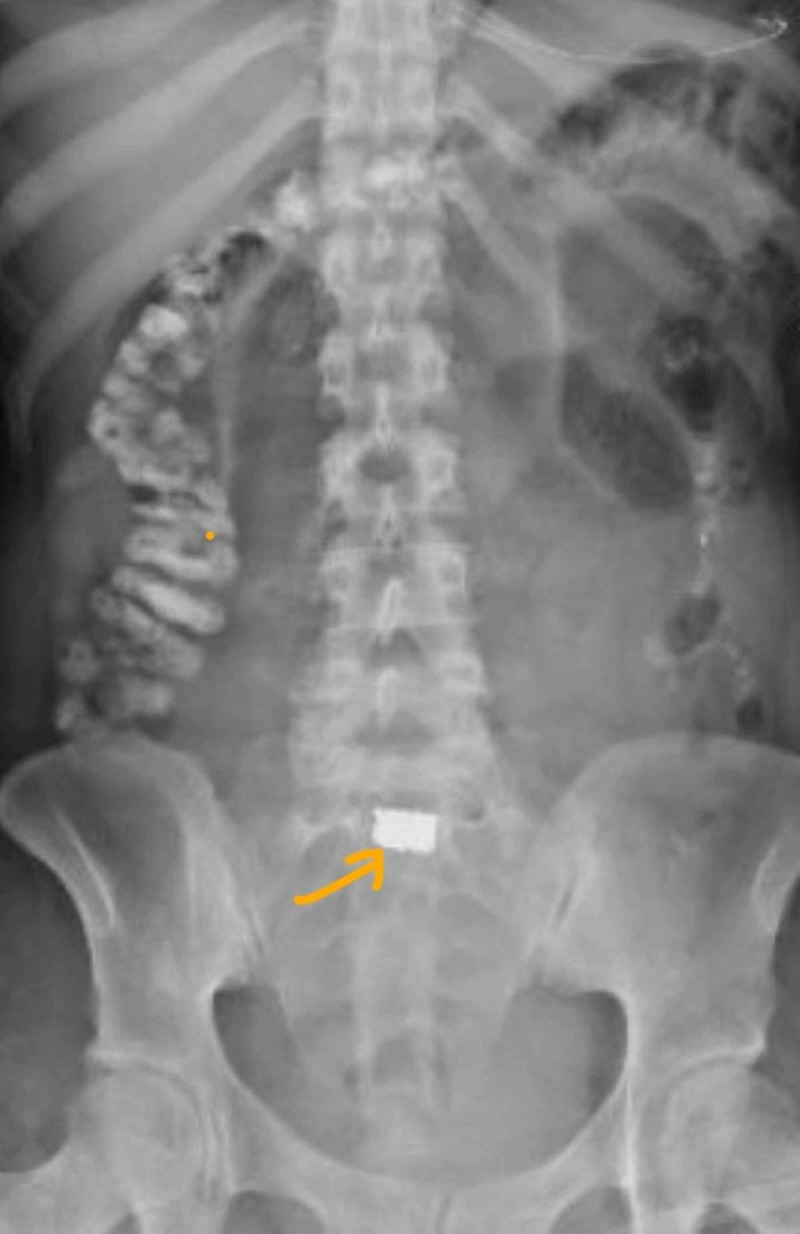
Bullet in the right lower quadrant for approximately four days

**Figure 5:**
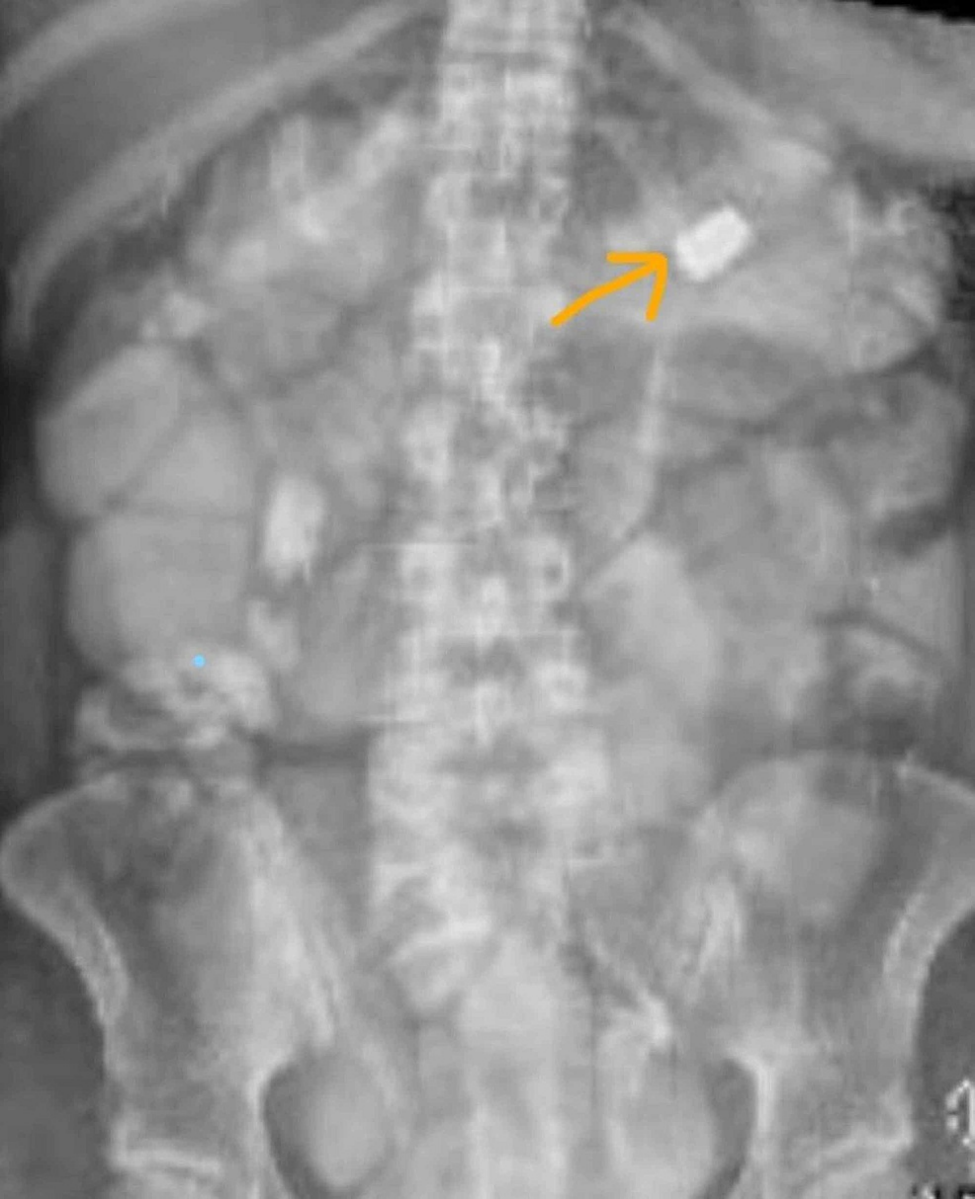
Progression in the position of the bullet, postoperative day 8

Postoperatively, once resuscitated, the patient had a return of neurological function with stabilization of base deficit on postoperative day 1. The patient had a high nasogastric tube bilious output with bloating despite passing flatus until postoperative day 3. A plain abdominal x-ray showed evidence of a bullet fragment within dilated loops of bowel. On the next day, the patient had bowel movement and relief of bloating only to reoccur the day after. Over the subsequent seven days, intermittent abdominal films showed a gradual progression of the bullet (Figures 2-5). During this time, we also performed an oral Gastrografin study; however, the patient had bowel movement only to have later bloating with vomiting. The abdominal films revealed that the patient had the bullet fragment in the right lower quadrant for approximately four days (Figure 4). On postoperative day 11, patient was able to pass the bullet and then have subsequent relief of her symptoms. There was no recurrence of symptoms after the passage of the bullet in the stool. The rest of the wounds healed appropriately with wound care.

## Discussion

In trauma, gunshot wounds are one of the most common trauma injuries to present to the emergency rooms [2]. In any trauma, a rapid systematic assessment of the patient with a primary survey concentrating on the airway, breathing, and circulation is needed [2-4]. For gunshot injuries to the back, a triple-phase computed tomography allows identifying if the injury is limited to retroperitoneum or is also involving intraperitoneal organs [2,3]. Intraperitoneal injuries identified on imaging mandate emergent exploratory laparotomy [1-4].

During exploratory laparotomy, it is essential to be cognizant of the lethal triad of hypothermia, acidosis, and coagulopathy [1-4]. Damage control surgery prioritizes handling the lethal triad by an abbreviated initial laparotomy and the subsequent definitive repair after achieving homeostasis [5-7]. Damage control laparotomy has shown to improve survival in patients [5-9]. A disadvantage is that it reduces the intraoperative time for the surgeon [5-9]. While there is evidence to support the extraction of foreign body bullets related to contamination of the colon to reduce infection postoperatively, most surgeons do not prioritize this in damage control emergency [10-13].

A foreign body fragment, if in a difficult location or not, causing immediate pressure effect doesn't need to be actively sought to save surgery time. Irrigation of wound with foreign bodies can help reduce infection [10-14].

In certain situations, as seen with our patient, a bullet fragment of a significant size can mimic the symptoms of gallstone ileus. Gallstones larger than 2.5 cm in size have shown to cause obstruction by getting lodged near the terminal ileum [15,16]. Gallstone ileus shows intermittent phases of abdominal pain, obstruction, resolution, and then obstruction again [15,16]. It needs an enterotomy to remove the mechanical obstruction at the distal ileum [16].

In this situation, the large bullet fragment mimicked her gallstone ileus by getting lodged in the distal ileum for approximately four days with intermittent obstruction. Here, it eventually passed; however, if the bullet had not passed, the patient would have needed a repeat laparotomy. Once the gallstone/foreign body is in the colon, it usually passes out with bowel movement [16].

## Conclusions

A large bullet left in the bowel loop can cause intraluminal obstruction by lodging in the bowel loops. The bullet fragment in this situation behaved like a gallstone ileus causing intermittent obstruction with relief. Whenever possible, bullet fragments should be extracted to avoid rare complications like in this case.

## References

[1] Lotfollahzadeh S, Burns B (2019). Penetrating Abdominal Trauma. StatPearls Publishing.

[2] Martin MJ, Brown CVR, Shatz DV (2019). Evaluation and management of abdominal gunshot wounds: a Western Trauma Association critical decisions algorithm. J Trauma Acute Care Surg.

[3] Carlson GL, Patrick H, Amin AI (2013). Management of the open abdomen: a national study of clinical outcome and safety of negative pressure wound therapy. Ann Surg.

[4] Caspers M, Schäfer N, Fröhlich M, Bauerfeind U, Bouillon B, Mutschler M, Maegele M (2018). How do external factors contribute to the hypocoagulative state in trauma-induced coagulopathy? - In vitro analysis of the lethal triad in trauma. Scand J Trauma Resusc Emerg Med.

[5] Malgras B, Prunet B, Lesaffre X (2017). Damage control: concept and implementation. J Visc Surg.

[6] Lamb CM, MacGoey P, Navarro AP, Brooks AJ (2014). Damage control surgery in the era of damage control resuscitation. Br J Anaesth.

[7] Mizobata Y (2017). Damage control resuscitation: a practical approach for severely hemorrhagic patients and its effects on trauma surgery. J Intensive Care.

[8] Roberts DJ, Zygun DA, Faris PD (2016). Opinions of practicing surgeons on the appropriateness of published indications for use of damage control surgery in trauma patients: an international cross-sectional survey. J Am Coll Surg.

[9] Dienstknecht T, Horst K, Sellei R, Berner A, Nerlich M, Hardcastle T (2012). Indications for bullet removal: overview of the literature, and clinical practice guidelines for European trauma surgeons. Eur J Trauma Emerg Surg.

[10] Rehman S, Slemenda C, Kestner C, Joglekar S (2011). Management of gunshot pelvic fractures with bowel injury: is fracture debridement necessary?. J Trauma.

[11] Quigley KJ, Place HM (2006). The role of debridement and antibiotics in gunshot wounds to the spine. J Trauma.

[12] Ordog GJ, Sheppard GF, Wasserberger JS, Balasubramanium S, Shoemaker WC (1993). Infection in minor gunshot wounds. J Trauma.

[13] Farooq A, Memon B, Memon MA (2007). Resolution of gallstone ileus with spontaneous evacuation of gallstone. Emerg Radiol.

[14] Sertkaya M, Emre A, Akbulut S, Vicdan H, Şanlı AN (2019). A typical gallstone ileus: clinical, radiological and operational findings. Turk J Gastroenterol.

[15] Ploneda-Valencia CF, Gallo-Morales M, Rinchon C (2017). Gallstone ileus: an overview of the literature [El íleo biliar: una revisión de la literatura médica]. Rev Gastroenterol Mex.

[16] Farkas N, Kaur V, Shanmuganandan A, Black J, Redon C, Frampton AE, West N (2018). A systematic review of gallstone sigmoid ileus management. Ann Med Surg.

