# Correction: The Tumbling Bullet: Subacute Intestinal Obstruction due to a Retained Bullet

**DOI:** 10.7759/cureus.c50

**Published:** 2021-10-22

**Authors:** Anupam K Gupta, Blake Edwards

**Affiliations:** 1 Minimally Invasive Surgery, University of Miami Hospital, Miami, USA; 2 General Surgery, Boca Raton Regional Hospital, Florida Atlantic University, Boca Raton, USA

Jorge A. Vega, originally listed as third author, has been removed from this article at his request. As reported by Dr. Vega and confirmed via investigation by relevant FAU faculty, Dr. Vega made no contribution to this manuscript nor was he aware of its submission or publication. As a result, we have removed Dr. Vega from the author list.

